# Effect of Binders on the Crushing Strength of Ferro-Coke

**DOI:** 10.3390/ma14040850

**Published:** 2021-02-10

**Authors:** Runsheng Xu, Shuliang Deng, Wei Wang, Heng Zheng, Shaopeng Chen, Xiaoming Huang, Fangfang Wang

**Affiliations:** 1 State Key Laboratory of Advanced Metallurgy, University of Science and Technology Beijing, Beijing 100083, China; 2 State Key Laboratory of Refractories and Metallurgy, Wuhan University of Science and Technology, Wuhan 430081, China; deng_shuliang@163.com (S.D.); wangwei74@wust.edu.cn (W.W.); mady016@wust.edu.cn (S.C.); 201902703096@wust.edu.cn (X.H.); 3 Chair of Ferrous Metallurgy, Montanuniversität Leoben, Franz-Josef-Straße 18, A-8700 Leoben, Austria; heng.zheng@stud.unileoben.ac.at; 4 School of Energy and Power Engineering, Beihang University, Beijing 100191, China; 5 Ningbo Institute of Technology, Beihang University, Ningbo 315800, China

**Keywords:** gas coal, iron ore powder, ferro-coke, crushing strength, binder

## Abstract

Ferro-coke, as a new burden of blast furnace (BF), can not only greatly reduce the energy consumption and CO_2_ emission, but also promote the resource utilization by using the low-quality iron ore and low-grade coal. However, the strength of ferro-coke decreased with the increasing amount of iron ore powder. In order to maintain the strength of ferro-coke while increasing the amount of iron ore powder, it is necessary to add binder during the coking process to enhance the strength of ferro-coke. In this paper, phenolic resin, silicon metal powder, corn starch, and coal tar pitch were used as binder for the fabrication of ferro-coke. I-type drum machine (I 600), scanning electron microscope (SEM), and X-ray diffraction (XRD) were applied to test the crushing strength, morphology, and microcrystalline structure of the ferro-coke. The results showed that the increasing amount of iron ore powder resulted in lower crushing strength, higher porosity, and the worse macroscopic morphology of ferro-coke. When the amount of iron ore powder reached 40%, obvious cracks appeared on the surface of ferro-coke. When the amount of iron ore was 30%, the crushing strength of ferro-coke dropped to 18.15%. Among the four binders, coal tar pitch could significantly enhance the cold crushing strength of ferro-coke through decreasing the porosity of ferro-coke and improving the bonding effect between carbon matrix particles. In the case of the 10% coal tar pitch addition, the cold crushing strength of ferro-coke was increased from 18.15% to 76.41%; meanwhile, its hot compression strength during gasification improved by 100N.

## 1. Introduction

Faced with the shortage of resources and the challenge of the environment, green growth has become a new indispensable development pattern [1]. The traditional blast furnace (BF) iron-making process has great demand for coke, which results in large CO_2_ emission. It has caused great pressure on the environmental governance, resource utilization, and economic development [2,3,4]. Ferro-coke, as a kind of high reactivity composite material, can reduce the initial gasification temperature of coke in a blast furnace. Thus, the temperature in the thermal reserve zone, the heat loss in the high temperature area, and the total fuel consumption per ton of hot metal can be reduced [5,6,7,8], which is of great significance to the energy conservation and emission reduction.

As the only solid burden in the high temperature area of BF, coke needs high strength to ensure the smooth operation of BF and maintain the smelting efficiency. However, the low strength limits the wide application of highly reactive ferro-coke. There are usually two ways to add iron into ferro-coke, namely material addition method and liquid phase adsorption method [9]. Seiji Nomura [10] added fine iron powder to the single coal and the mixed coals, respectively. When the addition amount of iron powder was 10%, the reactivity of ferro-coke increased from 38% to 58%; however, the crushing strength of ferro-coke decreased from 82% to 78%. Huixuan Zhang [11] added refined Canadian iron ore, Australia FMG iron ore (from Fortescue Metals Group Ltd), and Chinese iron ore into the same coal to fabricate ferro-coke. It was found that the reactivity of coke increased from 42.5% to 81% after 15% FMG powder addition, while the crushing strength (M_25_) was decreased from 89% to 79%. When the addition amount of FMG powder reached 20%, the reactivity of ferro-coke remained basically unchanged, and the crushing strength (M_25_) decreased to 58%. In addition, the reactivity and strength of ferro-coke obtained by liquid phase adsorption also changed because of the presence of iron. Hao Shi [12] used this method to prepare coke contained Fe_2_O_3_ with different mass fractions. When the amount of Fe_2_O_3_ was 5%, the initial gasification reaction temperature between coke and CO_2_ decreased from 843.8 to 617.13 °C, and the violent gasification reaction temperature decreased from 1250.82 to 1187.35 °C. The iron in the ferro-coke caused the earlier gasification between coke and CO_2_, resulting in the early degradation of coke. Furthermore, the post-reaction strength of ferro-coke also reduced. With the increase of iron content in ferro-coke, the strength decreased more obviously after reaction [10,11,12,13,14].

The low strength of ferro-coke restricts its extensive application in BF and the utilization of resources. The strength of ferro-coke can be improved by a hot briquette preparation process. The cold compressive strength of ferro-coke using hot briquette was more than 2000 N [15]. Hongtao Wang [16] increased the cold compressive strength of ferro-coke to 3490.89~4305.40 N by improving the preparation process. As reported, the strength of traditional coke can be improved by added binders [17,18]. Qiang Zhong [19,20] added coal tar pitch to industrial coal powder during coking; the drop strength and compressive strength of coke increased from 15.1 to 56.6 times/2 m and 0.45 to 13.06 MPa, respectively. Ataru Uchida [21] added hyper-coal (HPC), as a binder, into slightly-caking coal; the cold compressive strength of ferro-coke was increased significantly. Then, hyper-coal (HPC) and high fluidity hyper-coal (HF-HPC) were as two different binders added in the same coal; the results showed that HPC and HF-HPC could both enhance adhesiveness between coal particles. The HF-HPC had a stronger liquidity after soft melting, so that it had a greater promotion effect on the ferro-coke compressive strength than HPC [22,23]. In the previous studies, however, the addition amounts of iron ore powder were all less than 30%. Furthermore, most of them focused on the effect of iron ore on the performance of ferro-coke. Therefore, it is very important to clarify the effect of binders and the large amount of iron ore on the quality of ferro-coke.

In this paper, the addition amount of iron ore powder (10%, 20%, 30%, 40%, 50%) were used to reveal the effect of iron power amount on the crushing strength of ferro-coke. Then four types of binders (phenolic resin, silicon metal powder, corn starch, and coal tar pitch) were added into the coal to test their effect on the cold and hot crushing strength of ferro-coke.

## 2. Experimental Materials and Methods

### 2.1. Experimental Materials

**Figure 1:**
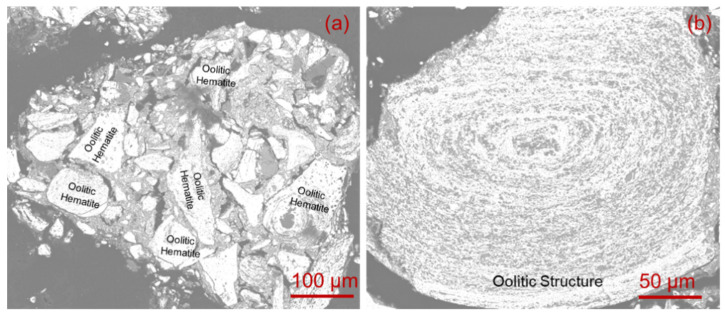
The SEM image of EX iron ore: (**a**) Oolitic hematite group; (**b**) Oollitic structure.

The raw materials of pulverized coal and iron ore used in the experiment were all from a Chinese steel plant. The pulverized coal is gas coal (QM), which is a slight caking coal. Its proximate analysis and ultimate analysis are analyzed based on GB/T 212-2008 and GB/T 31391-2015 [24,25,26,27]. The results of proximate analysis, ultimate analysis, and ash composition analysis of gas coal are shown in Table 1 and Table 2, respectively [28]. Table 3 shows the chemical composition of iron ore [28]. The iron ore is from Western Hubei (EX), the morphology of EX iron ore can be observed through SEM, as shown in Figure 1. The hematite in iron ore is obvious in form of concentric layered structure. The four kinds of binders are phenolic resin (refractory binder), silicon metal powder (inorganic binder), corn starch (biomass binder), and coal tar pitch.

### 2.2. Experimental Methods

**Figure 2:**
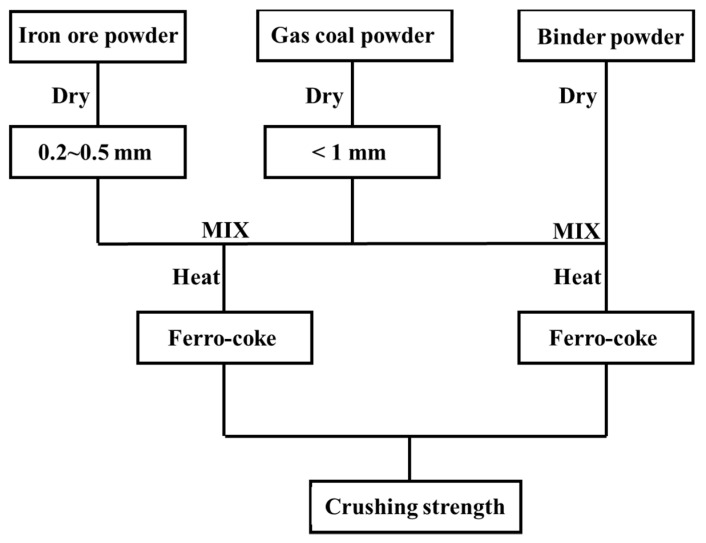
Experimental scheme.

The gas coal and iron ore were pulverized by laboratory crusher. The gas coal powder with particle size less than 1 mm and iron ore powder with particle size between 0.2 and 0.5 mm were obtained through screening. The obtained pulverized coal and iron ore powder were placed in a drying chamber and dried at 40 °C for 30 min. Three factors were considered in the experiment: the addition of iron ore powder, the type of binder, and the addition amount of binder. The weight of each sample is 10 g, and the blended ratios are listed in Table 4. Figure 2 is the flow chart of sample preparation. Firstly, ferro-coke was made by coal and iron ore powder. Following, the suitable proportion of iron ore powder was determined after crushing strength test. Afterwards, the best binder and its addition amount were determined to further increase the crushing strength of ferro-coke.

#### 2.2.1. Sample Preparation

**Figure 3:**
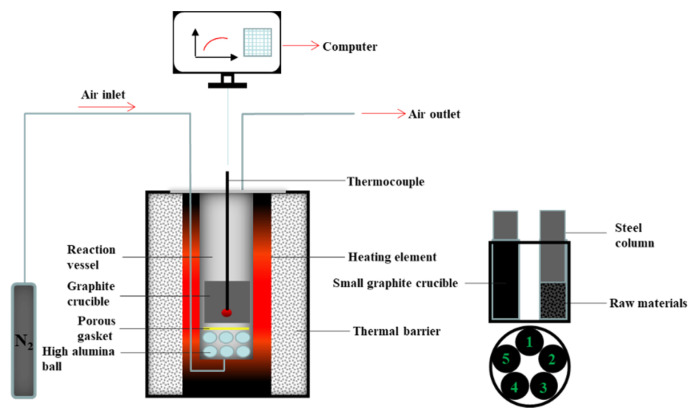
The diagram of self-developed vertical tubular furnace.

In the experiment of ferro-coke preparation, a self-developed vertical tubular furnace was used. The maximum temperature of the equipment could reach 1300 °C, and the gas, such as N_2_, CO_2_, CO, and water vapor, could be introduced. The type and flow of the gas could be controlled by the gas control cabinet during the experiment. The device schematic diagram is shown in Figure 3. The raw material was placed in a 20 mm diameter and 60 mm height cylindrical graphite crucible. A stainless steel column with a diameter of 20 mm and a height of 70 mm was pressed above the raw material. The steel column could provide about 6 kPa of pressure. These small graphite crucibles were placed in a large graphite crucible. The experiment used a three-stage heating program: firstly the temperature was increased from room temperature to 600 °C at 3 °C/min, then from 600 °C to 1100 °C at 5 °C/min, and finally the temperature was kept for 240 min. N_2_ of 1 L/min was injected throughout the processing until the sample temperature cooled to 170 °C.

#### 2.2.2. Determination of Crushing Strength of Ferro-Coke

The samples were taken out and weighed. The initial mass was marked as *m*_0_. The crushing strength test was carried out with I drum machine (Φ130 mm–H700 mm). The crushing time was 30 min and the rotation speed was 20 r/min. The sample was taken out after 30 min and the mass of the particles with a particle size of >10 mm was recorded as *m*_30_. The crushing strength index of ferro-coke can be expressed by the following formula, Equation (1):

(1) $$Crushing\;strength\;Index=\frac{m_{30}}{m_{0}}\times 100\%$$

where $m_{0}$ is the initial mass of the ferro-coke before crushing, and $m_{30}$ is the mass of the ferro-coke whose diameter is greater than 10 mm after crushing 30 min.

#### 2.2.3. Determination of Hot Compression Strength of Ferro-Coke

**Figure 4:**
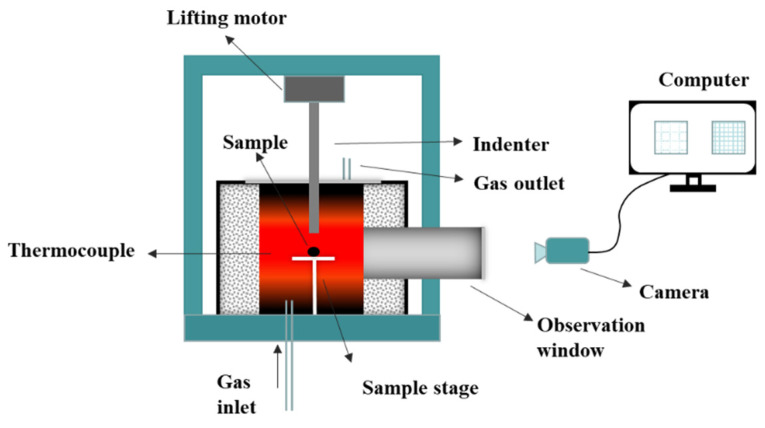
Testing equipment of hot compression strength of sample during gasification.

The hot compression strength testing equipment is shown in Figure 4. The samples were loaded in the sample stage at room temperature, and then heated to 1373 K at a heating rate of 10 K/min. Then, 1 L/min N_2_ was introduced to the equipment as a protection gas. After the sample stayed at 1373 K for 10 min, the protection gas was switched to gasification gas CO_2_ at 1 L/min. When the gasification reached to a certain time (0, 10, 20, 40, and 60 min), the hot compression strength was tested and recorded.

#### 2.2.4. Structure Analysis of Ferro-Coke

The ferro-coke obtained from the coking experiment was cut from the middle part, and the cross section was slightly grounded with sandpaper. The ferro-coke sample was vertically placed in a mold 20 mm high with 30 mm diameter. The cool inlay material was made up of a 2:1 ratio of epoxy resin and polyamide resin, which was still liquid at ambient temperature. The cool inlay material was evenly poured into the mold and solidified in the vacuum chamber, so that it could fully enter the inner pores of the ferro-coke. The cool inlay material worked as the framework of the ferro-coke during polishing to maintain the complete surface morphology of the ferro-coke. The ferro-coke samples were observed by an OLYMPUS DSX (Tokyo, Japan) optical microscope at a magnification of 69 times in the dark field, and the panoramic views of the cross-section were obtained using the superimposition (EFI) mode. The image J software was used to calculate the porosity of the ferro-coke. The ferro-coke was ground and then analyzed by X-ray diffraction (XRD, MAXima-7000, Shimadzu, Japan) that used Cu Kα radiation (λ = 1.5406 Å) with the scan rate of 2°/min in the 2 degree range of 10° to 90° [29,30].

## 3. Results and Discussion

### 3.1. Effect of Iron Ore Addition on the Crushing Strength of Ferro-Coke

**Figure 5:**
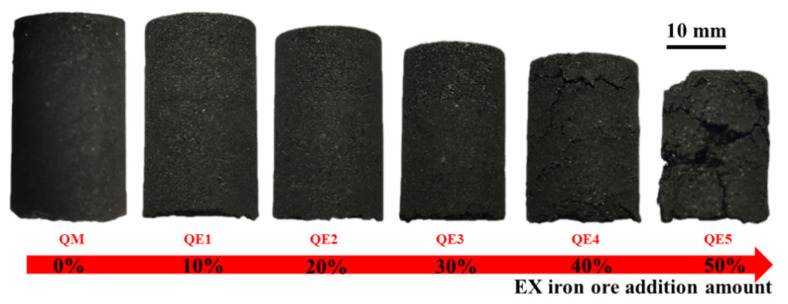
Macroscopic morphology of ferro-coke with different amounts of iron ore.

Figure 5 shows the comparison of macro morphology of ferro-coke with different amount of iron ore powder. The results showed that the surfaces of QM, QE1, and QE2 with 0%, 10%, and 20% addition were smooth and compact. When the amount of iron ore powder reached 30%, small pits appeared on the surface of QE3 and the structure was deteriorated. When the amount of iron ore powder was 40%, QE4 had obvious cracks. When the amount of iron ore powder reached 50%, QE5 was seriously damaged and the structure was loose. The marco morphology change rule obviously indicated that the strength of ferro-coke decreased with the iron ore amount increasing.

**Figure 6:**
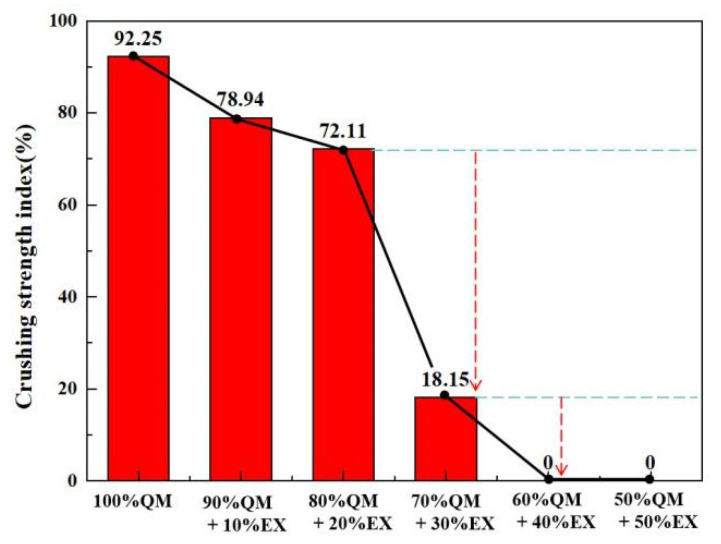
Crushing strength index of ferro-coke with different amounts of iron ore.

The crushing strength of ferro-coke with different amounts of iron ore is shown in Figure 6. Without iron ore, QM had the highest crushing strength, which was 92.25%. When the addition amounts of iron ore were 10% (QE1) and 20% (QE2), the ferro-coke could maintain a higher crushing strength, which were 78.94% and 72.11%, respectively. When the addition amount of iron ore was 30%, the crushing strength of QE3 dropped to 18.15%. The crushing strength of ferro-coke QE4 and QE5 were both 0. It confirmed that the crushing strength of ferro-coke decreased with the addition amounts of iron ore. When the amount of iron ore exceeded 30%, the ferro-coke could not maintain an enough strength for ironmaking.

**Figure 7:**
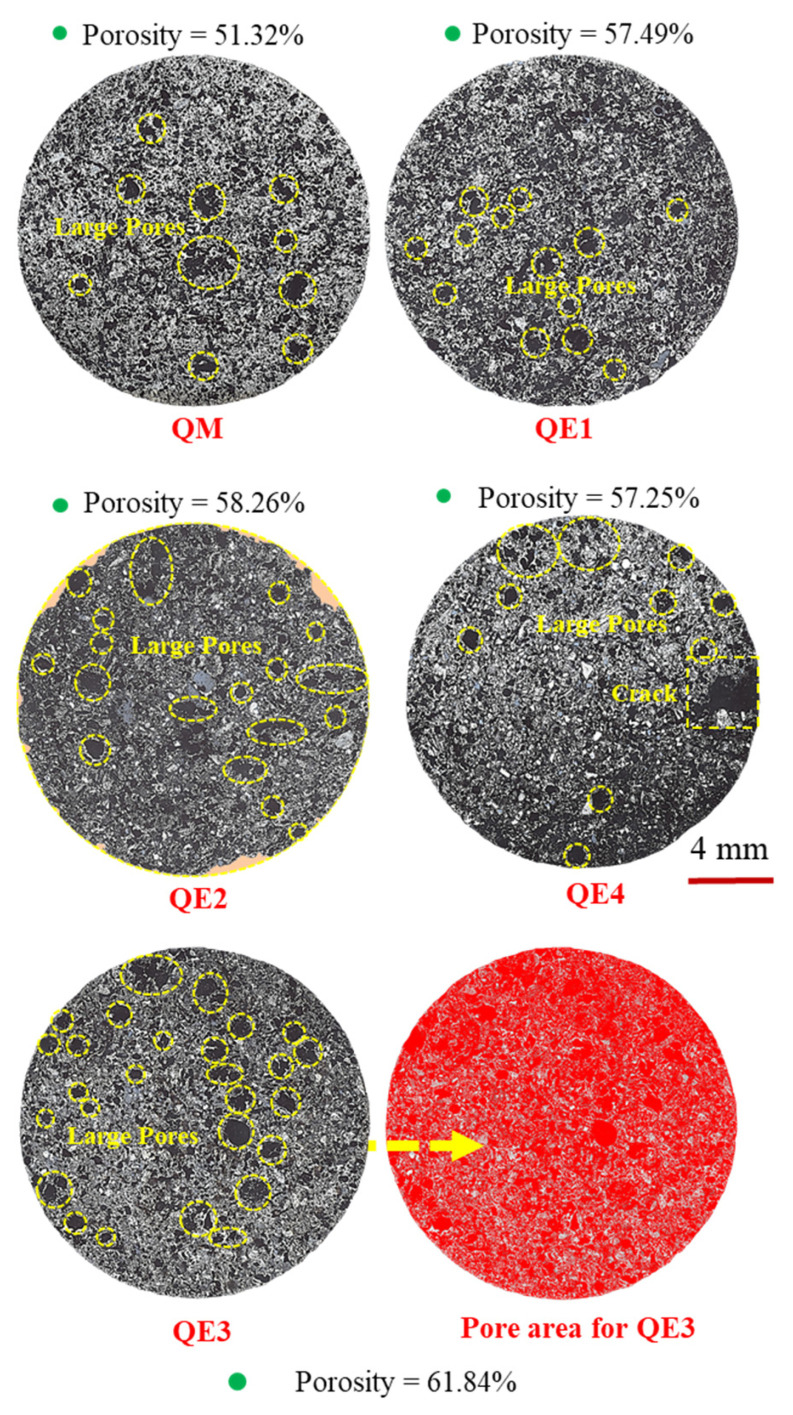
Panorama of ferro-coke on cross-section with different amounts of iron ore.

Figure 7 shows the panorama view of ferro-coke on cross-section with different amounts of iron ore. The panorama of QE5 was not shown due to it being broken. The pore area of ferro-coke is expressed as red part by Image J, and the red area ratio is used to represent the sample’s porosity in this study. The porosities of different ferro-cokes were 51.32% (QM), 57.49% (QE1), 58.26% (QE2), 61.84% (QE3), and 57.25% (QE4), respectively. It could be found that the porosity of ferro-coke was increased with the addition of iron ore. However, when the addition of iron ore was 40%, the porosity decreased to some extent, which may be caused by iron ore powder embedded in the pore [31].

**Figure 8:**
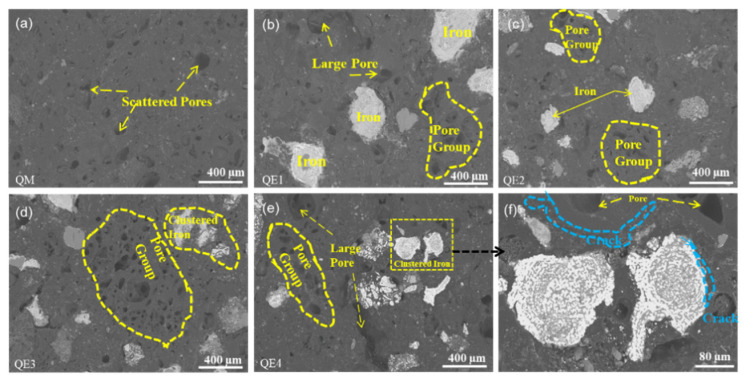
SEM of ferro-coke with different amounts of iron ore: (**a**) 100%QM; (**b**)90%QM + 10%EX; (**c**) 80%QM + 20%EX; (**d**) 70%QM + 30%EX; (**e**) 60%QM + 40%EX, and (**f**) 60%QM + 40%EX.

The microstructures of ferro-coke were compared to explain the strength difference, as shown in Figure 8. It could be clearly found that with the increase of iron ore addition, the pore sizes increased, and the amounts of pores and the reduced iron increased as well. Due to the incomplete bonding between iron particles and carbon matrix (as the blue dotted line showed in the part of QE4 in Figure 8), the migration of iron particles during the caking process would cut off the bonded carbon matrix and hinder the bonding between coal particles, so that the crushing strength of ferro-coke gradually decreased.

**Figure 9:**
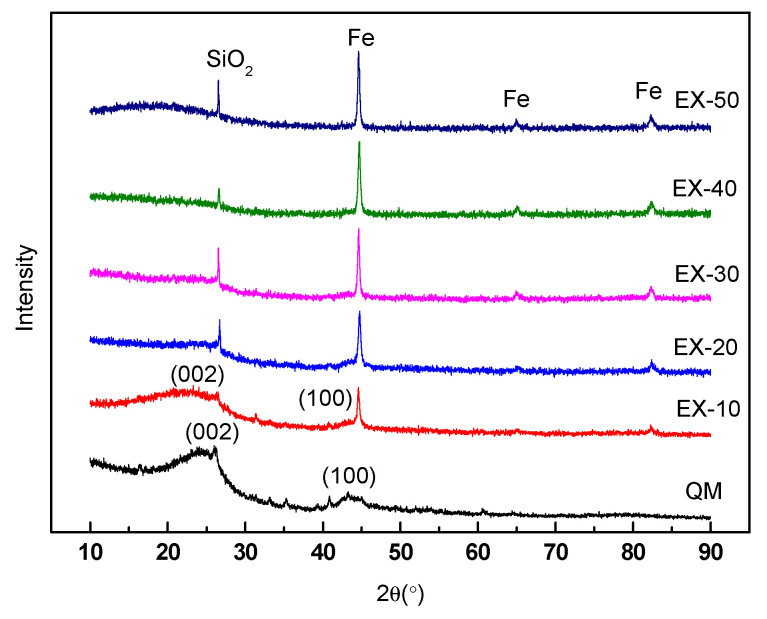
XRD of ferro-coke with different amounts of iron ore.

As shown in Figure 9, the peaks of Fe and SiO_2_ were ubiquitous in all ferro-cokes. The iron was reduced from iron ore during the caking process, and SiO_2_ mainly came from the gas coal. The addition of iron ore would lower the intensity of (002) peak and the (100) peak of ferro-coke. There were unobvious peaks of carbon (002) and carbon (100), when the addition amounts of iron ore was above 20%. The difference of peak type indicated the difference of the microcrystalline structure in the coke matrix. Generally, the better the degree of carbon microcrystalline structure, the higher the degree of graphitization of carbon matrix. These results revealed that the iron ore could affect the development of microcrystalline structure of coal during gasification. Because the Fe_2_O_3_ in iron ore were gradually reduced to Fe, some carbon molecules would participate in Fe_2_O_3_ reduction reaction rather than the development of microcrystalline structure [24]. However, the carbon matrix strength strongly related to its microcrystalline structure. The higher microcrystalline structure would benefit to the coke strength. In other words, the sharper peaks of (002) and (100), the crushing strength of coke would be higher [32,33,34]. Therefore, the decrease of crystallinity degree of carbon with the iron ore content increasing was also one of the main reasons leading to the decrease of cold crushing strength.

It could be concluded that the macroscopic morphology of ferro-coke would get worse with the addition of iron ore. The increase of the porosity and the worse bonding between coal particles would destroy the microcrystalline structure of ferro-coke, resulting in poor cold crushing strength of ferro-coke.

### 3.2. Effect of Different Binders on the Crushing Strength of Ferro-Coke

**Figure 10:**
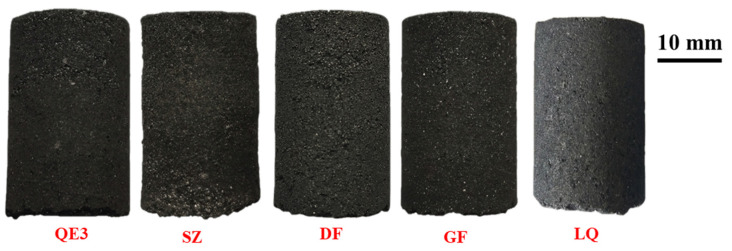
Macroscopic morphology of ferro-coke with different binders.

Different binders were added in the raw coal of QE3 as the coking scheme in Table 4. The macroscopic morphologies of the different ferro-coke were shown in Figure 10. The macroscopic morphology was varied with different binders. The surface of ferro-coke with phenolic resin (SZ) was uneven. The carbon matrix in the ferro-coke with corn starch (DF) was easy to break off and form small holes. A lot of silicon was embedded in the surface of ferro-coke added silicon powder (GF). The silicon fell off easily from the matrix because of the poor bond between silica powder and carbon matrix. The ferro-coke with coal tar pitch (LQ) had a fairly macroscopic morphology, but some large pores also appeared on the surface.

**Figure 11:**
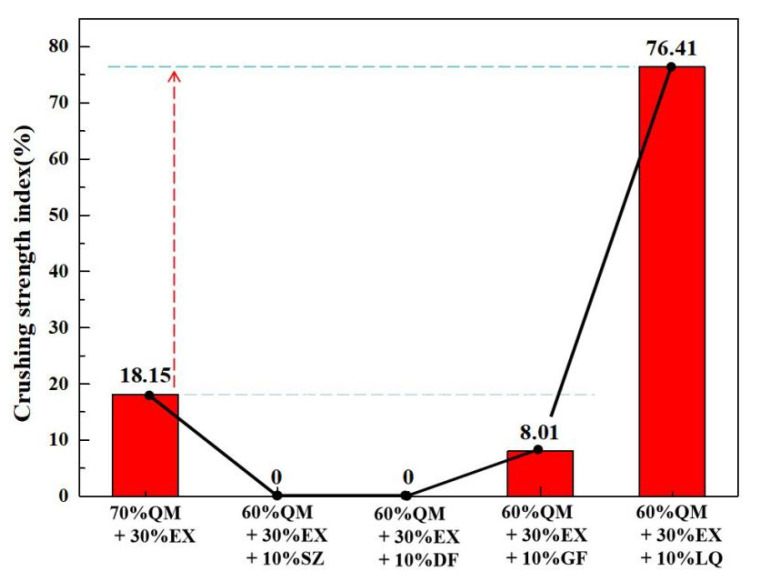
Crushing strength index of ferro-coke with different binders.

The effect of different binders on the cold crushing strength of ferro-coke is shown in Figure 11. Coal tar pitch showed significantly positive effect on the strength, which increased the crushing strength of ferro-coke from 18.15% to 76.41%. However, the crushing strength of ferro-coke GF, DF, and SZ was decreased in turn.

**Figure 12:**
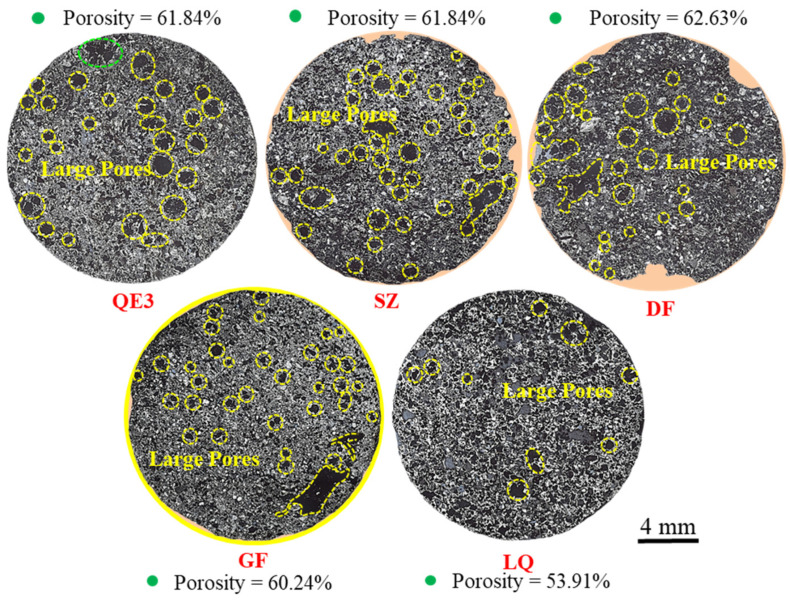
Panorama of ferro-coke on cross-section with different binders.

The porosity of ferro-coke with different binders was analyzed by image J software, which was 60.12% (SZ), 62.63% (DF), 60.24% (GF), and 53.91%(LQ), respectively. It can be found that the porosity of LQ was decreased significantly compared to QE3, while that of other ferro-cokes (SZ, DF, and GF) increased to a certain extent. As shown in Figure 12, the carbon matrix fell off from the cross-section of SZ, DF, and GF (red dotted lines). The orange area in the Figure 12 is the broken part of the ferro-coke matrix, which is filled with the resin material.

**Figure 13:**
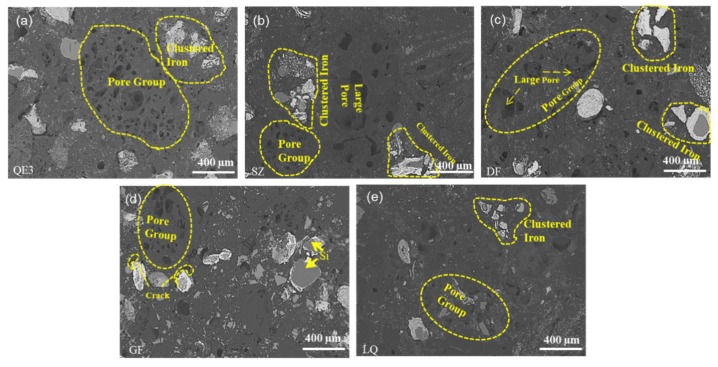
SEM of ferro-coke with different binders: (**a**) 70%QM + 30%EX; (**b**) 63%QM + 27%EX + 10%SZ; (**c**) 63%QM + 27%EX + 10%DF; (**d**) 63%QM + 27%EX + 10%GF, and (**e**) 63%QM + 27%EX + 10%LQ.

Figure 13 shows the SEM images of ferro-coke with different binders. It could be found that, in SZ and DF, the small pores connected with each other to form a large pore leading to the large interspace between iron particles and carbon matrix. There are a large amount of Si and iron particles in the GF carbon matrix, which lead to the poor bond of coal particles and the low crushing strength of GF. Compared with QE3, the number and diameter of large pores in LQ decreased, which resulted in its decrease of porosity and increase of crushing strength.

**Figure 14:**
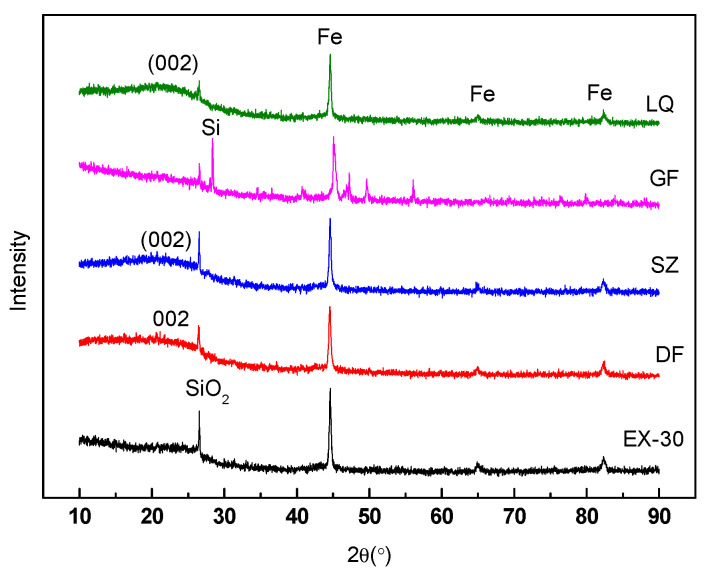
XRD of ferro-coke with different binders.

From the XRD of ferro-coke with different binders in Figure 14, it could be found that the microcrystalline structure was varied. Compared with QE3, the microcrystalline structure of SZ, DF, and LQ became ordered. However, the cold crushing strength of SZ and DF were still low, which indicated the greater influence factors of cold crushing strength was the ferro-coke’s porosity. The highest peak of Si in the XRD of GF revealed that the silicon powder could not react with C to generate SiC at 1100 °C, so it had no bonding effect on the coal particles. Previous study also showed that the strength was acquired by sintering at 1100–1200 °C in a reduction atmosphere for metallic silicon powder, and under that situation, the formed nanofibrous SiC would enhance the strength of the matrix [35]. For phenol resin, the temperature was much higher (about 1200–1300 °C). In order to let the phenol resin be useful, the basic or acidic catalyst should be added [36]. For corn starch, it could only benefit the strength of raw briquette, but could not improve the bonding strength of iron coke, because the corn starch would break down at high temperatures, and because the coal tar pitch had similar molecular structure as coal and could form a large number of liquid phases (colloidal) at high temperature, so it could strongly improve the wetting and bonding between raw materials, subsequently enhancing the strength of ferro-coke. It could be concluded that the coal tar pitch was the only one useful binder to enhance the cold crushing strength under the traditional coking temperature (1000 °C) in this study.

### 3.3. Effect of Coal Tar Pitch Amount on the Crushing Strength of Ferro-Coke

**Figure 15:**
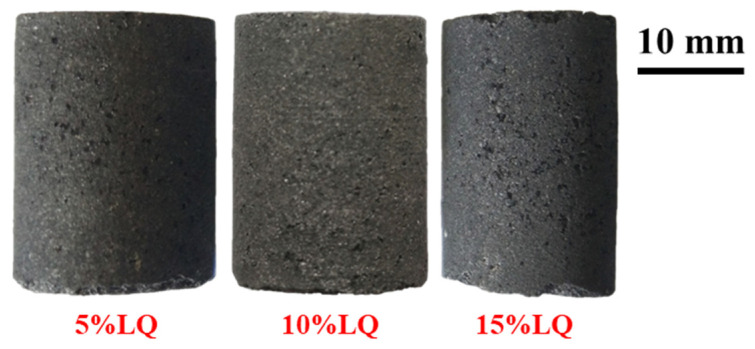
Macroscopic morphology of ferro-coke with different amounts of coal-tar pitch.

Different amounts of coal tar pitch in the raw coal of QE3 were used to explore the optimal addition amount of coal tar pitch. It could be found that large pores appeared on the surface of all the ferro-coke, as shown in Figure 15, and the 5%LQ and 15%LQ have more pores than 10%LQ.

**Figure 16:**
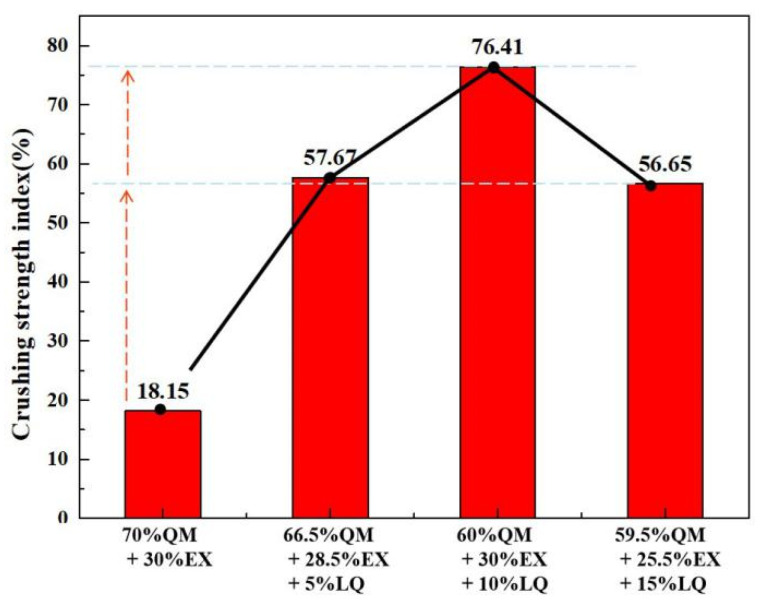
Crushing strength index of ferro-coke with different amounts of coal tar pitch.

The crushing strengths of ferro-coke with different amounts of coal tar pitch are shown in Figure 16. It could be found that the crushing strength of ferro-coke increased first and then decreased with the increasing content of coal tar pitch. When the addition amount was 10%, the crushing strength was the highest.

**Figure 17:**
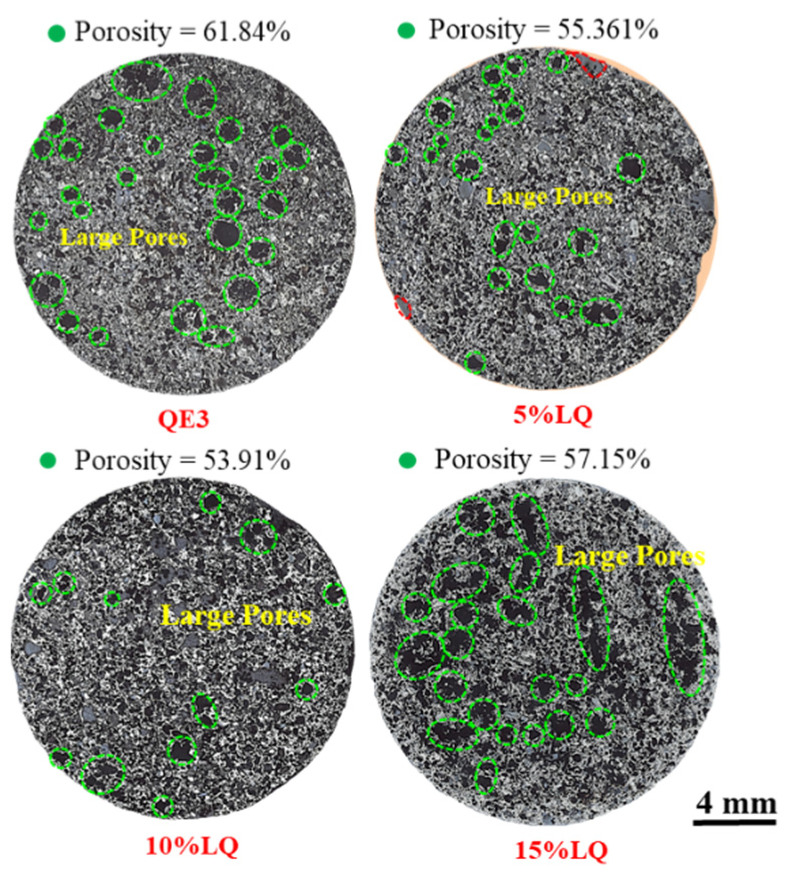
Panorama of ferro-coke on cross-section with different amounts of coal-tar pitch.

Figure 17 shows the panoramas of ferro-cokes with different addition amount of coal tar pitch. The porosities of ferro-coke with coal tar pitch were 55.36% (5%LQ), 53.91% (10%LQ), and 57.15% (15%LQ), respectively. These porosities were lower than that of QE3, but the porosity of ferro-coke decreased first and then increased with the increasing of coal tar pitch. In addition, there were more large pores in 5%LQ and 15%LQ than in 10%LQ. This phenomenon was determined by the amount of coal tar pitch in raw materials. During the carbonization of raw materials, the coal tar pitch would produce many liquid phases to help the bonding of coal particles. However, this positive effect depended on the amount of produced liquid phased. When the amount of liquid was little, the coal powder bonding efficient was not enough. When the amount of liquid was too much, it was easy to form a porous structure. These above two situations were not conducive to the strength of coke, so the addition amount of coal tar pitch had a suitable content. Through this study, it could be concluded that 10% coal tar pitch was the best for improving the crushing strength of ferro-coke. 

**Figure 18:**
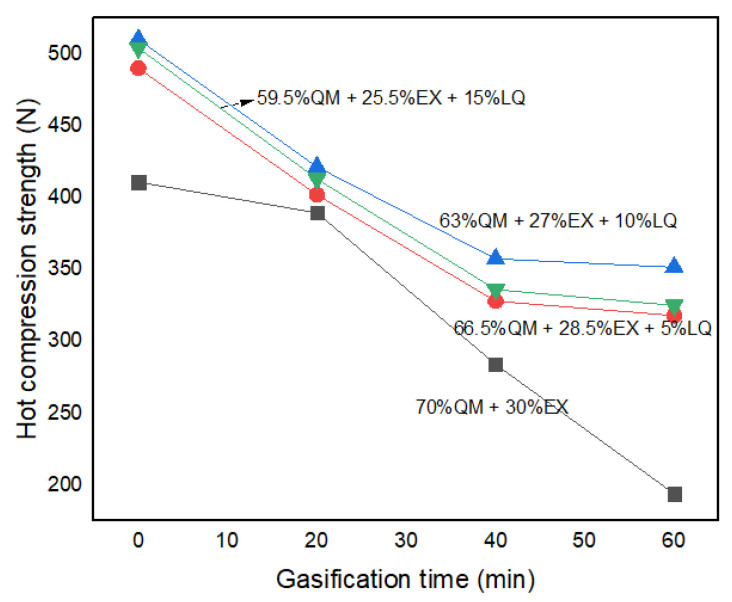
Hot compressing strength of samples during gasification.

In order to check the effect of LQ binder on the hot compression strength of ferro-coke during gasification, the hot compression strengths of ferro-cokes with different amount of LQ binder were tested. Figure 18 shows that the hot compression strength of all samples gradually decreased with the gasification time increasing. It revealed that the gasification had a strong effect on the hot compression strength due to the carbon consumption. It also would be found that the LQ binder would obviously improve the hot compression strength of ferro-coke, and the improved effect was best in the case of 10%. It must be pointed out that the best compression strength of ferro-coke after 60 min gasification was 351.6 N, which was much less than that of traditional coke (1298.2 N) [28]. It is well known that ferro-coke will charge with sinter/pellets to improve the reduction efficient at the upper areas of blast furnace, and it is expected to be fully consumed in the bulk layer. Therefore, ferro-coke does not have the skeleton role as traditional coke, so it does not need the same high thermal strength as traditional coke. In further study, the suitable hot compression strength values for different kinds of blast furnace will be determined. At the same time, more efficient binders to improve the ferro-coke’s strength of both cold and hot states will be discovered so as to further guide the preparation of ferro-coke.

## 4. Conclusions

The following conclusions can be obtained by investigating the effect of iron ore powder and binders on the macroscopic morphology, microstructure, and crushing strength of ferro-coke:

(1) The cold crushing strength of ferro-coke decreased with the increasing amount of iron ore. When the addition amount of iron ore was 20%, the cold crushing strength of ferro-coke was 72.11%. However, when the amount reached 30%, the cold crushing strength of ferro-coke dropped to 18.15%.

(2) Different binders had different influence on the crushing strength of ferro-coke. Coal tar pitch could increase the cold crushing strength from 18.15% to 76.41%. Other binders (phenolic resin, corn starch, and silicon power) showed negative effect on the crushing strength of ferro-coke.

(3) The porosity of ferro-coke with coal tar pitch decreased obviously, and the large pores kept away from each other. Compared with 5%LQ and 15%LQ, 10%LQ contained less pores and showed the highest cold crushing strength. In addition, the 10% LQ binder would improve the hot compression strength of ferro-coke by about 100N. In further study, more effective binders to improve the ferro-coke’s strength of both cold and hot states should be explored.

## Figures and Tables

**Table 1:** Property analysis of QM coal (wt.%) [28].

Sample	Proximate Analysis, Air Dry Basis				Ultimate Analysis, Air Dry Basis				
	Moisture	Volatiles	Ash	Fixed Carbon	C	H	N	S	O
QM	2.63	31.58	8.27	57.52	69.78	8.07	0.73	0.19	10.33

**Table 2:** Chemical composition of QM coal ash (wt.%) [28].

Sample	SiO_2_	Al_2_O_3_	Fe_2_O_3_	CaO	MgO	TiO_2_	SO_2_	K_2_O	Na_2_O	MnO
QM	67.53	21.97	1.57	3.02	0.60	1.47	0.91	1.76	0.75	0.02

**Table 3:** Chemical composition of the EX iron ore (wt.%) [28].

Sample	TFe	FeO	CaO	SiO_2_	MgO	Al_2_O_3_	P	S	LOI
EX	55.15	0.82	0.19	11.59	0.34	4.55	0.13	0.02	1.63

(LOI: Loss on Ignition).

**Table 4:** Raw material ratios for the preparation of different kinds of ferro-cokes (wt.%).

Binders							Phenolic Resin	Corn Starch	Silicon Power	Coal Tar Pitch		
Sample	QM	QE1	QE2	QE3	QE4	QE5	SZ	DF	GF	LQ	5%LQ	15%LQ
Amounts of coal	100	90	80	70	60	50	63	63	63	63	66.5	59.5
Amounts of iron ore	0	10	20	30	40	50	27	27	27	27	28.5	25.5
Amounts of binder	0	0	0	0	0	0	10	10	10	10	5	15

## Data Availability

The data presented in this study are available in insert article.
